# Contrast enhanced computed tomography of small ruminants: Caprine and ovine

**DOI:** 10.1371/journal.pone.0287529

**Published:** 2023-12-21

**Authors:** Juliette M. Caffrey, Patricia K. Thomas, Susan E. Appt, Heather B. Burkart, Caitlin M. Weaver, Michael Kleinberger, F. Scott Gayzik

**Affiliations:** 1 Biomedical Engineering, Wake Forest University School of Medicine, Winston-Salem, NC, United States of America; 2 Pathology–Comparative Medicine, Wake Forest University School of Medicine, Winston-Salem, NC, United States of America; 3 Army Research Directorate, DEVCOM Army Research Laboratory, Aberdeen Proving Ground, MD, United States of America; Ethicon Endo Surgery, UNITED STATES

## Abstract

The use of small ruminants, mainly sheep and goats, is increasing in biomedical research. Small ruminants are a desirable animal model due to their human-like anatomy and physiology. However, the large variability between studies and lack of baseline data on these animals creates a barrier to further research. This knowledge gap includes a lack of computed tomography (CT) scans for healthy subjects. Full body, contrast enhanced CT scans of caprine and ovine subjects were acquired for subsequent modeling studies. Scans were acquired from an ovine specimen (male, Khatadin, 30–35 kg) and caprine specimen (female, Nubian 30–35 kg). Scans were acquired with and without contrast. Contrast enhanced scans utilized 1.7 mL/kg of contrast administered at 2 mL/s and scans were acquired 20 seconds, 80 seconds, and 5 minutes post-contrast. Scans were taken at 100 kV and 400 mA. Each scan was reconstructed using a bone window and a soft tissue window. Sixteen full body image data sets are presented (2 specimens by 4 contrast levels by 2 reconstruction windows) and are available for download through the form located at: https://redcap.link/COScanData. Scans showed that the post-contrast timing and scan reconstruction method affected structural visualization. The data are intended for further biomedical research on ruminants related to computational model development, device prototyping, comparative diagnostics, intervention planning, and other forms of translational research.

## Introduction

The use of small ruminants, mainly young sheep and goats, in translational research studies is increasing. Small ruminants are desirable for use as animal models due to their similarities in dimension, physiology, and anatomy to humans [1]. The age equivalence relationship between these animals and humans is well-defined making them ideal for translational research [2]. Small ruminants demonstrate reasonable longevity, low costs, are easy to handle, and raise few ethical concerns [2]. Furthermore, they are commercially available, gentle, clean, and easy to maintain [3, 4].

Sheep and goats are used for research in diseases of the cardiovascular, respiratory, urinary, osteoarticular, integumentary, and reproductive systems, as they have a large array of anatomic and physiologic similarities to humans [1]. For example, in the cardiovascular system both sheep and goats have been successfully used in preclinical studies for cardiac transplant, valve replacement, and stents [4, 5]. Goats are used to study atrial fibrillation due to their large hearts and congenital myotonia, which is a condition seen in humans and fainting goats [6, 7]. Sheep have the same respiratory rate, air flow, and resistance as humans, making them an ideal model for studies focusing on the respiratory system, particularly asthma and more complex diseases such as cystic fibrosis [8, 9]. Additionally, pre-term and full-term lambs have the same lung structure as prenatal and neonatal babies. These similarities include the branching of the respiratory tree and submucosal gland composition, making sheep an ideal model for studying infant respiratory conditions [10]. In orthopedics, small ruminants are used for studies spanning rheumatoid arthritis, fracture repair devices, and intervertebral disk replacement [11–15]. The composition of their bones, metabolism, bone size, joint size, ratio of cartilage to subchondral bone, bone remodeling, and axial loading of the cervical spine is similar to humans, making them an ideal model for bone [16–23]. Goats have easy to access joints, along with subchondral joints that are more similar to humans than sheep, dogs, and rodents [16]. The similar sizing of sheep to humans results in good reproducibility in surgical interventions and imaging information when translated to humans [24].

Given the array of uses for these animals for biomedical research, they are naturally also used in the development of computational models. Such models, the geometry of which are typically constructed from computed tomography (CT) scans, can be thought of as “digital twins” of the animals themselves. Models can be validated with existing data and, provided they are used within the bounds of previous validation, can be used to generate additional data in the absence of additional experimentation. “Digital twin” animal models have been used have been used in many previous studies to investigate a wide range of scenarios. These are certainly not limited to medium to large ruminants. Arora et al. used MRI scans to develop a FEM of a monkey brain to study TBI [25]. McCarty et al. used micro-CT of the metacarpophalangeal joint of standardbred horses to study impact stress during mid stance loading [26]. Hinterhofer et al. used CT scans of the left hind foot of a dairy cow to study the effects of different flooring on the stress and strain in the limb [27]. Ruminants have also been used in such research. Mazoochian et al. used CT and micro-CT imaging to develop a finite element model (FEM) of an ovine hip [28]. Gibbons et al. used post-mortem CT scans of an ovine subject to develop a solid mechanics model of an adult sheep utilizing dynamic Lagrangian based finite elements for the evaluation of injury biomechanics in behind armor blunt trauma (BABT) impact scenarios [29]. Tests for various countermeasure designs can thus conceivably be done entirely *in-silico*. Still others have utilized ruminant models for fluid dynamics approaches focused on understanding heat stress on animals [30]. While this list is far from exhaustive, it demonstrates the utility of making such imaging protocols and the images themselves available to the biomedical research community since the development of computational models requires high quality medical imaging of the subject. Such modeling applications provide valuable insights while reducing the need for additional animal studies.

While small ruminants make an excellent animal model for research, the translation of this research to humans requires standardization of scaling for results [31–33]. A disadvantage in using small ruminants as an animal model is the lack of data available in the literature and large variability between studies such as breed, surgical interventions, disease state, and treatment [1]. The lack of standard practice when using these animal models makes it difficult to compare results across studies [1]. An important aspect of this knowledge gap includes the lack of CT scan data for these ruminants. CT scans are a valuable tool for medicine and research that allow for visualization of nearly all body parts to be used in monitoring, diagnosis, and planning for surgical intervention [34]. For small ruminants, CT diagnostics have been used for the reproductive tract, gastrointestinal tract, lymph nodes, and brain [35, 36]. CT scans are also commonly used in research studies involving small ruminants, especially with preclinical orthopedic treatments and computational modeling [25, 37–39]. Despite the wide use of CT scans there are no baseline full body scans of sheep or goats available in the literature.

The objective of this study therefore is to acquire full body CT image datasets of a sheep and goat to serve as baseline image data for translational research using ruminants. Given the use of these animals in studies of the circulatory system, baseline and contrast enhanced data is provided. Data discussed in this work are available for download through the form located at: https://redcap.link/COScanData.

## Methods

Whole body CT scans of sheep and goats with and without contrast were acquired for this study. This study was conducted in compliance with the Animal Welfare Act and other Federal statutes and regulations. The study adhered to the principles stated in the Guide for the Care and Use of Laboratory Animals, Institute for Laboratory Animal Research (1996) and was approved by the Wake Forest Institutional Animal Care and Use committee with written consent (A20-161). The United States Army Medical Research and Development Command Office of Research Protections Animal Care and Use Review Office also approved this study with written consent (W911NF2120034). All procedures were performed under the supervision of collaborating veterinarians (Appt, S.E., DeLoid, H.B.).

A female Nubian goat (30–35 kg) and a male Katahdin sheep (30–35 kg) were acquired at approximately one year of age (May Family Enterprises, Buffalo Mills, PA). Animals were Q-fever tested before delivery. After arrival, animals were housed together in a sand paddock. In order to prevent the transfer of zoonotic diseases, all personnel who handled the subjects were required to wear appropriate personal protective equipment including gloves, gowns, and masks. Subjects were transported to the scanner in individual crates.

### Scan protocol

**Fig 1:**
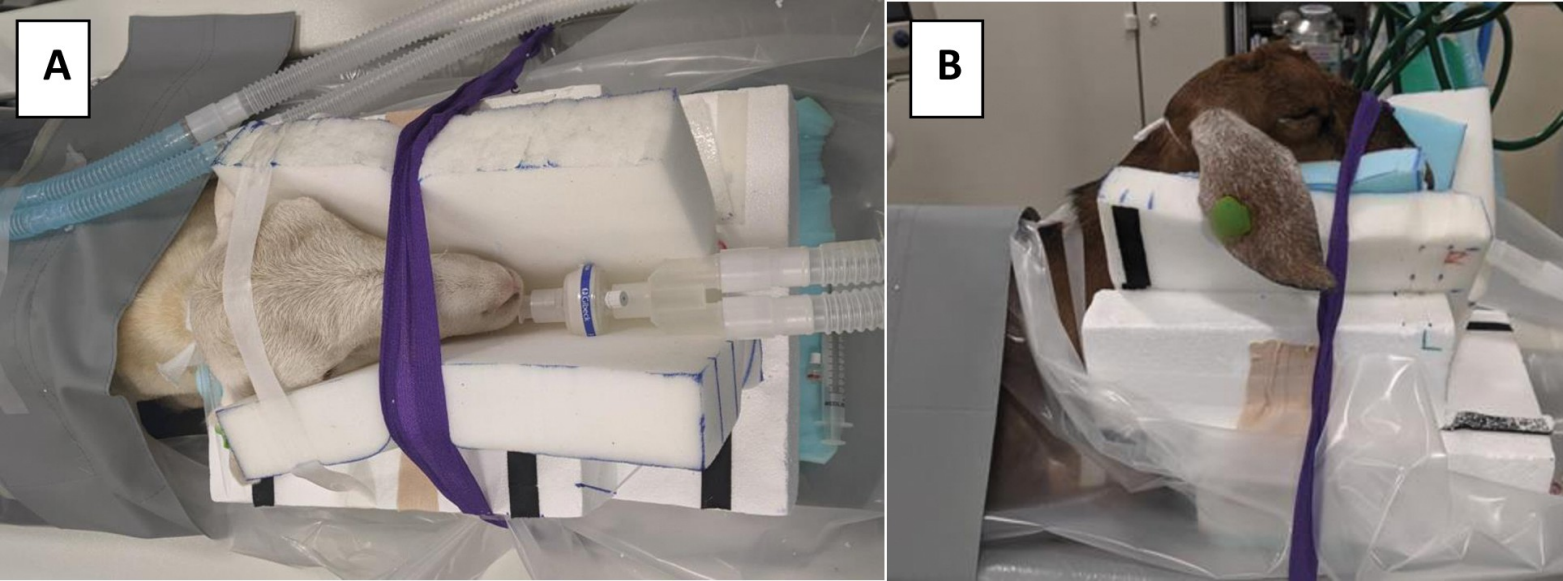
Image of the head support used for positioning. A. Top view showing channel that holds the breathing tube. B. Lateral view showing neck alignment.

Animals were fasted for 24–48 hours prior to scans in order to prevent bloating or regurgitation during anesthesia. Ketamine [10mg/kg IM] and Dexmedetomidine [0.01mg/kg IM] were administered for sedation. Atropine [0.02 mg/kg IM] was given to the goat to reduce salivary secretions and prevent bradycardia. Prior to moving to the scanning bed a 20G IV catheter was placed in the left or right cephalic vein for contrast administration and an endotracheal tube was inserted to allow maintenance of anesthesia with isoflurane gas. The anesthetic gas was administered for the duration of the scan procedure. Vital signs (rectal temperature, heart rate, respiratory rate, and SpO_2_) were recorded approximately every 10 minutes. Subjects were positioned in sternal recumbency. During the positioning process the subjects were wrapped in a sheet of plastic to protect the scanner from bodily fluids. The subjects’ heads were supported using a foam head support, seen in Fig 1, to keep the head at the proper angle for the breathing tube and to attempt to maintain alignment of the spine. Once in the desired position, straps were used to secure the subjects to the table.

The scans were taken using a Siemens SOMATOM Definition Flash CT scanner located at the Wake Forest Baptist Health Clarkson Campus, CTAWP98633. The scans used a wedge-3 filter, an exposure time of 500 ms, a slice thickness of 0.6 mm, a pixel size of 0.9 mm, and matrix of 512 x 512 pixels per slice. Each subject underwent a scout image prior to being scanned to determine the location of the subject on the table. Scans were taken using a radiation level of 100 kV and 400 mA. Scans were taken with no contrast, 20s post contrast, 80s post contrast, and 5 min post contrast. 1.7 mL/kg of Omnipaque 350 contrast was delivered at a rate of 2 mL/s using a power injector. Each scan was reconstructed for bone (B60f, sharp) and soft tissue (B30f, medium smooth).

Following scanning, animals were removed from anesthesia for recovery and Atipamezole [0.1 mg/kg IM] was administered. Once recovered, animals were returned to the paddock. Animals used in this study were released to local farms through established adoption channels. While the data presented are intended for general biomedical research use, the specific purpose for collecting this data by the investigators is for future finite element (FE) model development for *in-silico* BABT studies.

## Results

All CT scan data listed in Table 1 are available for download in DICOM format. Data can be acquired through the form located at: https://redcap.link/COScanData.

**Table 1:** Scans collected.

Subject	Contrast	# Images in Series	Recons	Scan Name
Ovine Subject	None	1947	Soft Tissue	S_NC_ST
			Bone	S_NC_B
	20s	1941	Soft Tissue	S_20s_ST
			Bone	S_20s_B
	80s	1941	Soft Tissue	S_80s_ST
			Bone	S_80s_B
	5 min	1941	Soft Tissue	S_5m_ST
			Bone	S_5m_B
Caprine Subject	None	1963	Soft Tissue	G_NC_ST
			Bone	G_NC_B
	20s	1963	Soft Tissue	G_20s_ST
			Bone	G_20s_B
	80s	1963	Soft Tissue	G_80s_ST
			Bone	G_80s_B
	5 min	1963	Soft Tissue	G_5m_ST
			Bone	G_5m_B

**Fig 2:**
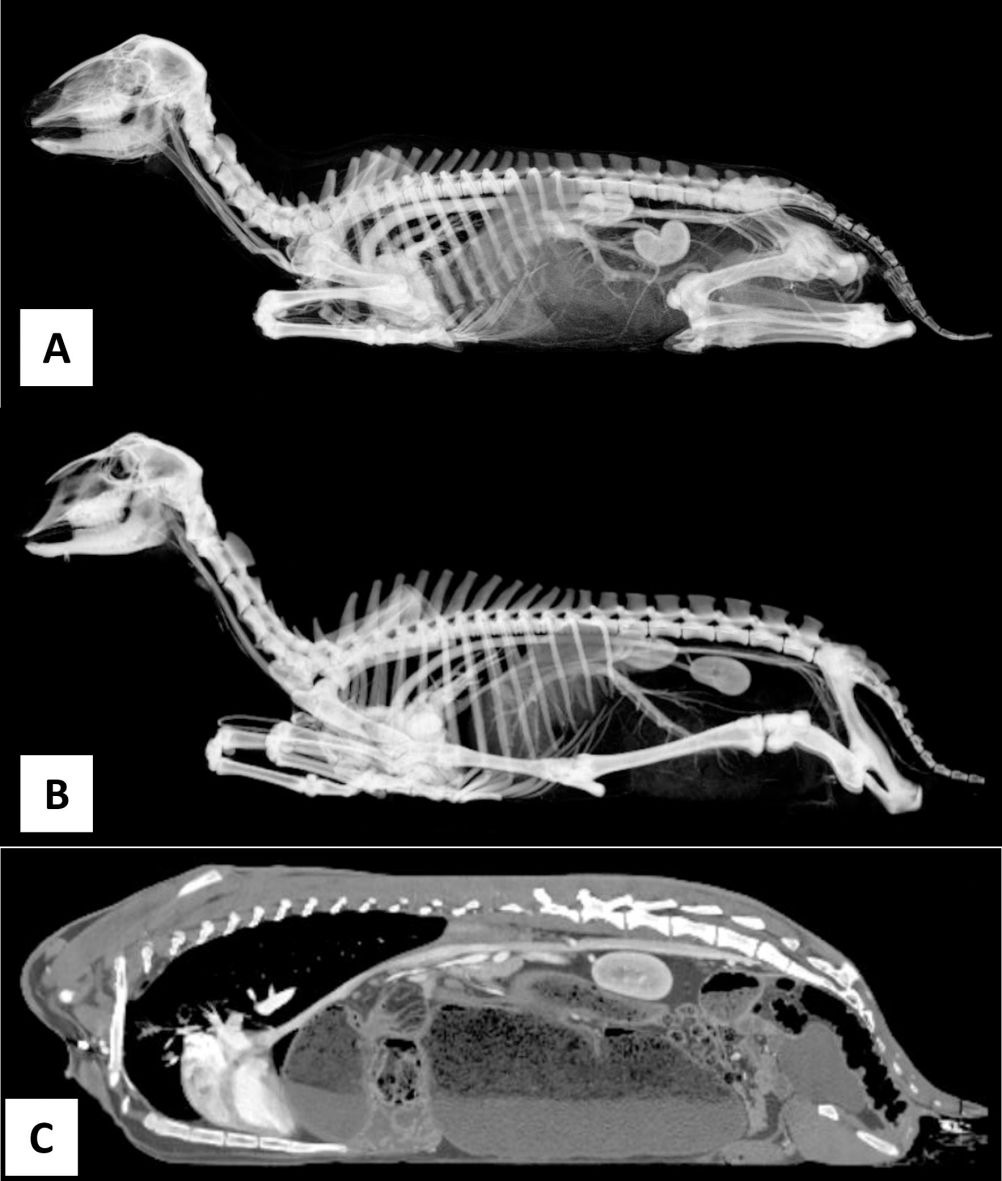
A sagittal 3D view of the sheep (A) and goat (B) using a lumbar filter. (C) A sagittal 2D view of the goat. Images taken from the 20s post contrast scans with soft tissue reconstruction.

**Fig 3:**
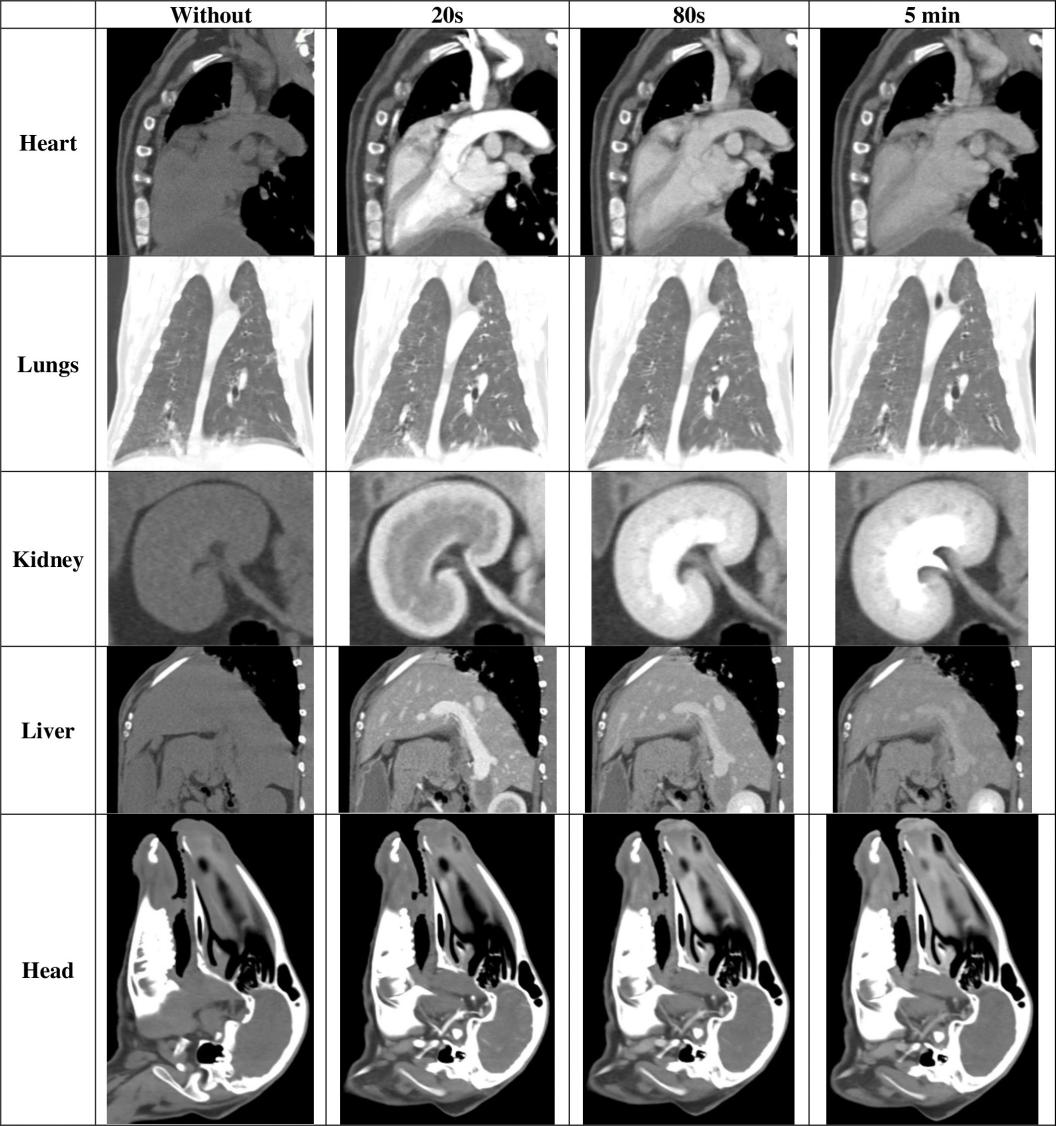
Visibility of select organs for each scan time point taken from soft tissue reconstructions of the sheep scans. The lung images were taken using a lung window to highlight the details inside the lungs, all other images were taken using the default.

Each subject took about one and a half hours to anesthetize, position, scan, and regain consciousness. Both subjects remained fully anesthetized and immobile for the durations of the scans. There were no allergic reactions to the medications delivered. No atypical geometry was noticed in the scans for any of the subjects (Fig 2). The visibility of the internal structures was affected by the use and timing of contrast (Fig 3).

In the without contrast scans, typical soft tissue contrast for organs with respect to surrounding structures are identified and the bone is the only structure with high intensity. Contrast enhanced scans show a trend from arterial, to venous, to urinary system progression of contrast. At 20 seconds post-contrast, the soft tissue structures are most easily identified including the heart and major blood vessels. At 80 seconds post-contrast, the contrast begins to fade in the arterial system whereas in the urinary tract the contrast level continues to build. At 5 minutes post-contrast the urinary track is highly visible but the contrast has been mostly metabolized throughout the rest of the body. The lungs and the head are not affected by contrast, but the vasculature within them is most visible 20 seconds post-contrast.

**Fig 4:**
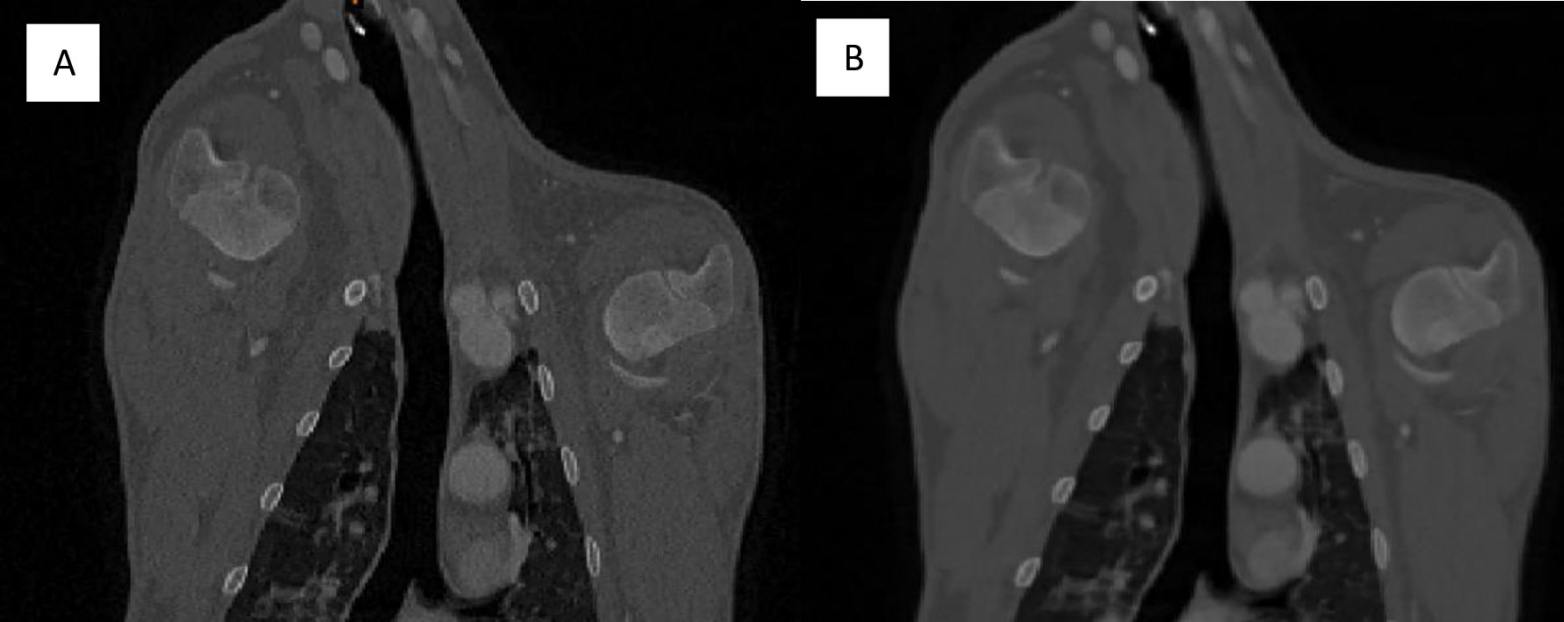
Coronal slice 279 of the without contrast sheep scan. A. Bone reconstruction. B. Soft tissue reconstruction.

The visibility of various structures is also affected by the reconstruction of the image stack post scanning. The comparison between a bone reconstruction (B60f sharp) and a soft tissue reconstruction (B30f medium) taken from the sheep without contrast scan is shown in Fig 4.

The bone reconstruction increased the sharpness of the scan, making cortical shell of bones easier to identify, but also increased pixilation of the image. The soft tissue reconstruction decreased pixilation making the appearance of soft tissue structures more consistent in intensity level. The decreased pixilation also makes the images appear slightly blurrier, particularly in the bones.

**Fig 5:**
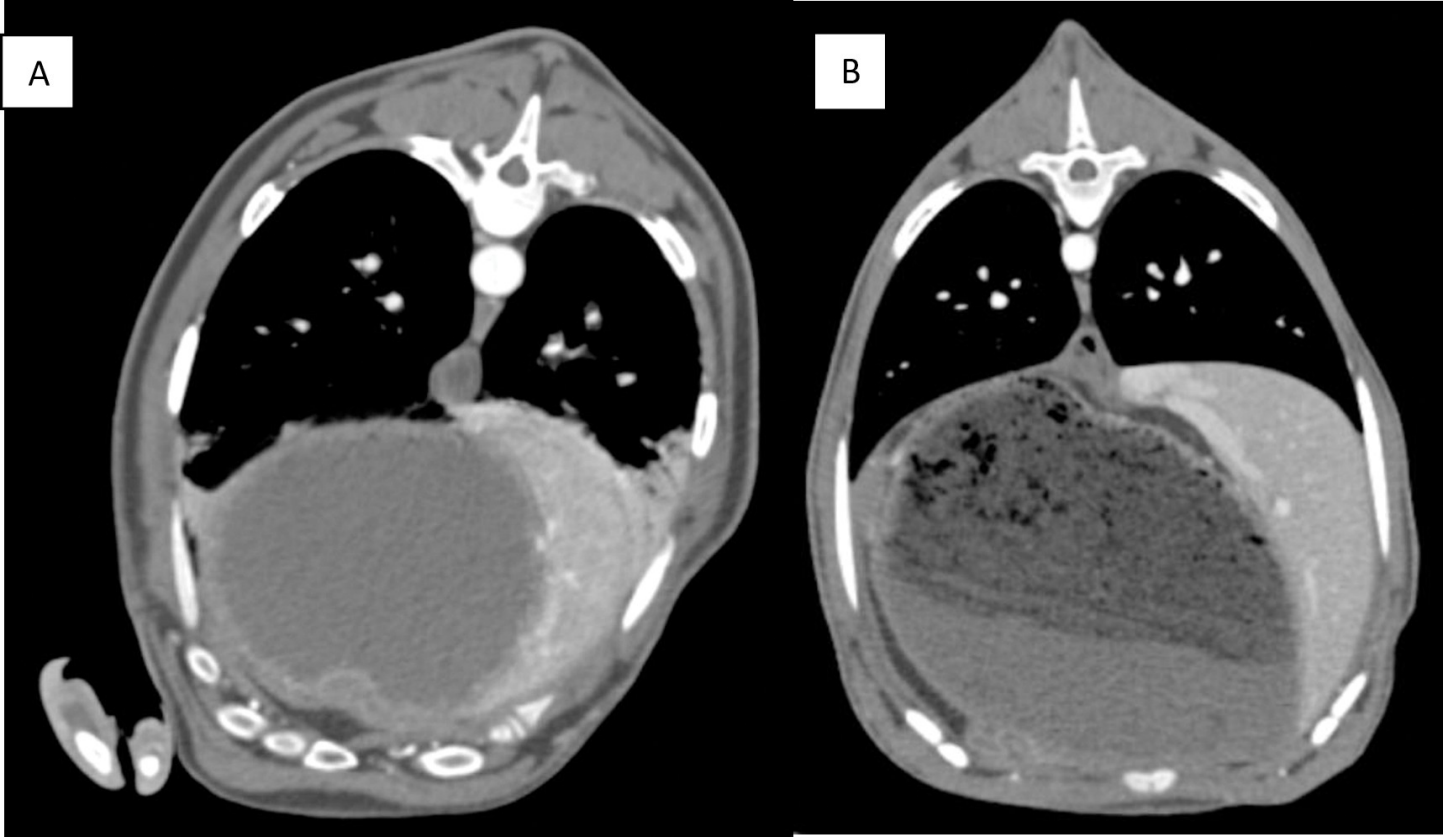
Axial cross section of the sheep (A) and the goat (B) at the T9 vertebral level. Images taken from the 20s post contrast scan using a soft tissue reconstruction.

While sheep and goats are generally considered to be very similar animals anatomically, when comparing the CT scans of these two images some differences were observed. A major difference between these animals is the shape of their torsos, the goat becomes narrowed on its dorsal side and has a more prominent spine than the sheep (Fig 5).

In addition to the overall torso shape, it can also be seen that goats have a sharper boundary between their lungs and abdominal cavity. At the T9 vertebral level the aortic diameter of the sheep is 16 mm and the goat is 13 mm, the percentage of the torso that is taken up by the abdominal cavity is 60% in the sheep and 67% in the goat, and the goat torso is 48% larger than the sheep.

## Discussion

The image data that accompany this technical brief will serve as a helpful reference for sheep and goat anatomy for a myriad of purposes in biomedical research. Given the use of CT data in research, this may vary from computational model development to device prototyping, to anatomical training, to comparative diagnostics to healthy specimens, and beyond. This reference of typical geometry will also help provide a common point for comparison between studies and as baseline imaging for translational research. Additionally, the structures visible in these scans can be segmented for use in FE model development. This image data will also be helpful for future studies developing scanning protocols related to the visibility of specific structures at various time points post contrast administration.

After sedation, the subjects lost conscious muscle contraction to hold their spinal alignment, which needed to be supported through low density foam supports. The goats were easier to align than the sheep due to the prominence of the vertebral spinous processes, allowing for the alignment to be palpated during the positioning process. Imperfections in spinal alignment may cause FEM developers to run spine straightening simulations to manually adjust the model’s positioning after segmentation.

The findings of this study show that the visibility of individual structures vary based on contrast timing and reconstruction methods. Bones and calcified cartilage are best viewed in bone reconstruction scans and are not affected by contrast. However, when using bone thresholding, it is best to use a bone reconstruction without contrast so that structures highlighted by contrast are not included in the Hounsfield unit threshold. The airway should be viewed in a soft tissue reconstruction and is not affected by contrast. Lungs are best viewed in a soft tissue reconstruction and are also not affected by contrast; however, using a scan at 20 seconds post contrast will make the blood vessels inside of the lungs and the boundary with the heart easier to discern. The heart is best viewed in a soft tissue reconstruction at 20 seconds post contrast. The boundary has motion artifact due to beating during the scan; cardiac gating can be used to improve heart scans however this was considered beyond the scope of this study. Visceral organs should be viewed in a soft tissue reconstruction at 80 seconds post contrast. The urinary track should be viewed in a soft tissue reconstruction at 5 minutes post contrast. Muscle and subcutaneous fat are not as easily viewed due to low relative contrast in any scan but are best viewed in a soft tissue reconstruction. The diaphragm cannot be directly viewed in CT scans. Since these scans were taken of live animals, breathing throughout the scans resulted in slight changes in locations of structures such as the ribs, diaphragm, and lungs in the different scans (different times post-contrast). Based on the pixel size anatomical details on the scale of 1 mm can be resolved. Vasculature becomes definable at a diameter of about 3 mm which is after the third bifurcation of the vasculature tree.

The selection of caprine and ovine species was based on their usefulness as animal models in translational research, however, there are some zoonotic concerns related to working with these species. The primary concern being Q Fever, which is contagious to humans and all subjects should be tested upon acquisition and handled with the appropriate personal protective equipment. This study looked at research breed subjects, or animals that were selectively mated for their genetic traits to form a population with very little genetic and anatomical variability. With that in mind, there will still be variations between individual subjects of these same breeds especially in subjects of differing age and sex, and it is important to note that there is a lack of research breed goats available. The use of subjects that are not bred for research increases variability in the study population. Additional factors that increase anatomical variation in subjects, which were not included in the study population, such as breed variation, increased aging, and sex cannot be accounted for in the referenced CT scan data. Future work could expand upon this study by using a cohort to capture the anatomical differences based on breed, age, and sex, or to align research with the breed presented in this work.

In conclusion, this study presented a protocol for scanning ruminants, including a representative image set of one ovine and caprine subject. For each subject, the image set includes scans taken with no contrast, 20s post contrast, 80s post contrast, and 5 min post contrast and reconstructed using bone and soft tissue windows. The image sets were reviewed by a veterinarian to confirm that they were representative of the breed for the age and gender scanned. The images provide sufficient detail for gross anatomical analysis and segmentation down to approximately the 1 mm scale. This data is intended to be used in future work for biomechanical studies. Through a brief review of literature, it was identified that the data could be used in studies related to computational model development, device prototyping, anatomical training, and scan protocol development.
